# Reduction in preterm birth rates during and after the COVID‐19 lockdown in Queensland Australia

**DOI:** 10.1111/ajo.13538

**Published:** 2022-05-17

**Authors:** Brittany Jasper, Tereza Stillerova, Christopher Anstey, Edward Weaver

**Affiliations:** ^1^ Department of Obstetrics and Gynaecology Sunshine Coast University Hospital Sunshine Coast Queensland Australia; ^2^ School of Medicine Griffith University Brisbane Queensland Australia; ^3^ North West Anglia Healthcare NHS Trust Cambridgeshire UK; ^4^ Department of Obstetrics and Gynaecology Cairns Hospital Cairns Queensland Australia; ^5^ Faculty of Medicine University of Queensland Brisbane Queensland Australia

**Keywords:** premature birth, stillbirth, lockdown, coronavirus, pandemic, influenza

## Abstract

**Background:**

Preventative strategies for preterm birth are lacking. Recent evidence proposed COVID‐19 lockdowns may have contributed to changes in preterm birth.

**Aims:**

To determine the prevalence of preterm birth and birth outcomes during and after the COVID‐19 lockdown at the Sunshine Coast University Hospital and the overall state of Queensland, Australia.

**Methods:**

Retrospective cohort analysis of all births in Queensland including the Sunshine Coast University Hospital, during two epochs, April 1–May 31, 2020 (lockdown) and June 1–July 31, 2020 (post‐lockdown), compared to antecedent calendar‐matched periods in 2018–2019. Prevalence of preterm birth, stillbirth, and late terminations were examined.

**Results:**

There were 64 989 births in Queensland from April to July 2018–2020. At the Sunshine Coast University Hospital, there was a significantly higher chance of birth at term during both lockdown (odds ratio (OR) 1.81, 95% CI 1.17, 2.79; *P* = 0.007) and post‐lockdown (OR 2.01, 95% CI 1.27, 3.18; *P* = 0.003). At the same centre, prevalence of preterm birth was 5.5% (30/547) during lockdown, compared to 9.1% (100/1095) in previous years, a 40.0% relative reduction (*P* = 0.016). At this centre during lockdown, emergency caesareans concurrently decreased (*P* < 0.01) and instrumental vaginal births increased (*P* < 0.01). In Queensland overall, there was a nonsignificant decrease in the prevalence of preterm birth during lockdown.

**Conclusions:**

There is a link between lockdown and a reduction in the prevalence of preterm birth on the Sunshine Coast. The cause is speculative at present, although increased influenza vaccination rates, decreased transmission of infections, and improved air quality may have been favourable in reducing preterm birth. Further research is needed to determine a causal link.

## INTRODUCTION

Preterm birth (PTB) is a major problem worldwide and is the leading cause of death in children under five.^1^ Despite advances in obstetric care, there has been no sustained reduction in the prevalence of PTB and preventative measures remain only partially successful.

**Figure 1:**
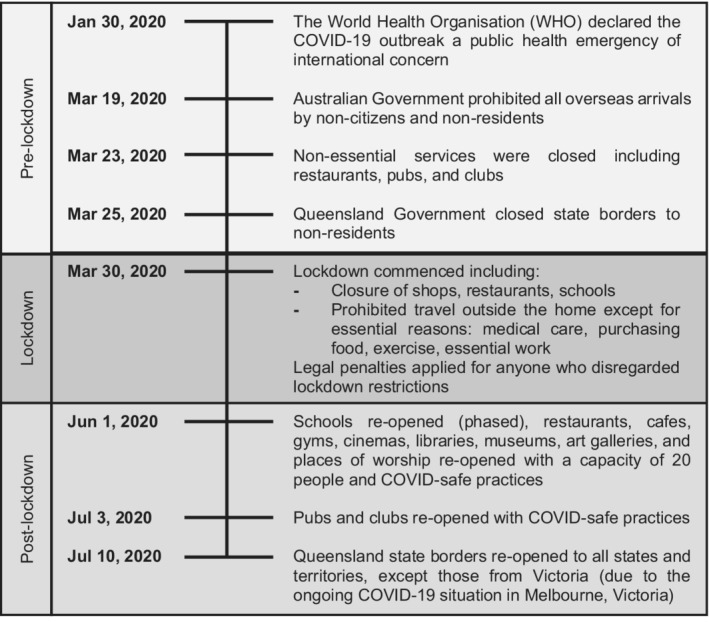
Timeline of COVID‐19 restriction implementation in Queensland, Australia.^3^

The World Health Organization declared COVID‐19 a pandemic on 11 March, 2020.^2^ This was followed by lockdowns across the globe, causing unprecedented effects on society and human behaviour in ways not seen before. In Queensland, Australia, the restrictions to mitigate the spread of COVID‐19 culminated in a nine‐week state‐wide lockdown from March 30 to June 1, 2020, with a phased easing of restrictions.^3^ During lockdown, all shops, restaurants, schools, universities, and non‐essential workplaces were closed.^3^ Residents were prohibited from leaving their homes except for essential reasons (Fig. 1).^3^

To minimise the risk of virus transmission, models of antenatal care at the Sunshine Coast University Hospital (SCUH) were adapted.^4^ Women were risk‐triaged to either virtual or in‐person appointments utilising COVID‐19 safe practices.^4^ While lockdown in Queensland has since lifted, restrictions continue to change rapidly throughout Australia in response to COVID‐19 cases.

In the wake of the pandemic, research emerged demonstrating the varied impact of lockdown on the prevalence of PTB worldwide.^5^, ^6^, ^7^, ^8^, ^9^, ^10^, ^11^, ^12^, ^13^, ^14^ Our primary aim was to determine the prevalence of PTB during and after the COVID‐19 lockdown at SCUH and the overall state of Queensland, Australia. Our secondary aims were to investigate birth outcomes including stillbirth and late termination of pregnancy, and compare perinatal characteristics, mode of delivery, and proposed aetiologies for preterm birth at SCUH during and after lockdown.

## METHODS

### Study population

This study focused on pregnant women in two geographical locations in Australia. Firstly, the state of Queensland, which services the health needs of over 5 million people, and secondly, SCUH, which provides care for the 400 000 people living in this area of Queensland.^15^ SCUH is a level five neonatal centre and cares for infants ≥29 weeks gestation or ≥1000 g birth weight. Of pregnant women in Queensland, 3.0% were aged less than 20 years and 4.0% were aged over 40 years,^16^ 7.5% identified as Aboriginal or Torres Strait Islander,^16^ 21.0% were obese,^17^ and 11.0% smoked during pregnancy.^17^

### Ethics approval and data collection

This study was reviewed by The Prince Charles Hospital Human Research Ethics Committee and approval was granted as a low or negligible risk project (Project ID 70040; November 3, 2020). Data pertaining to SCUH was collected from the health service birth registry. Queensland state‐wide data was sourced from Perinatal Data, Department of Health.

### Study design

Retrospective cohort analysis of births in Queensland, Australia was carried out during two epochs, April 1–May 31, 2020 (lockdown) and June 1–July 31, 2020 (post‐lockdown), compared to calendar‐matched periods from the preceding two years. There was a gap in the existing literature lacking analysis of birth outcomes after restrictions easing, thus a post‐lockdown period was included. The prevalence of birth outcomes including PTB, stillbirth, and late terminations were examined. All PTB less than 37 weeks gestation were analysed, including sub‐group analyses of extreme preterm (20 + 0–27 + 6 weeks), very preterm (28 + 0–31 + 6 weeks), and moderate‐to‐late preterm (32 + 0–36 + 6 weeks). To facilitate comparison of birth data at SCUH to Queensland overall, and to wholly represent all births after 20 weeks, no births were excluded because of multiple births, stillbirths after 20 weeks, or terminations after 20 weeks. At SCUH, perinatal characteristics were collected, including maternal age, body mass index (BMI), parity, and mode of delivery, proposed aetiology of birth outcomes including spontaneous PTB, iatrogenic PTB, stillbirth, and late termination. We subclassified spontaneous PTB into preterm premature rupture of membranes, preterm labour, and multiple pregnancy. Subclassifications for iatrogenic PTB included intrauterine growth restriction, hypertension, multiple pregnancy, fetal distress, placental abruption and antepartum haemorrhage, and other maternal comorbidities.

### Statistical analysis

All data were deidentified prior to analysis. Univariate logistic regression was performed to identify potential predictors of preterm or term birth. Variables with *P* < 0.20 were included in multivariate logistic regression analysis. The prevalence of PTB, stillbirth, and late terminations were calculated. Percent change was calculated to assess for difference between time periods. Descriptive statistics were reported as mean and standard deviation for normally distributed continuous data, median and interquartile range (IQR) for non‐normally distributed data, and frequencies and percentages for categorical data. Continuous data were tested for normality using the Shapiro–Wilk test and analysed using the *t*‐test. Categorical data were analysed using either a Mann–Whitney *U*‐test, χ^2^, or Fisher's exact test. *P*‐values < 0.05 were considered significant. All analyses were conducted using R statistical software.

### Patient and public involvement

Patient and public involvement began in the early stages of planning with the Preterm Infants Parents Association (PIPA), a registered Australian charity for families with premature babies. The PIPA team reviewed the study design and research questions. A plain language summary will be distributed to the PIPA network.

## RESULTS

There were 64 989 births in the state of Queensland, Australia, including 3296 births at SCUH between April 1–July 31, 2018–2020. Univariate logistic regression of births at SCUH revealed maternal age, BMI, and parity were nonsignificant predictors of PTB. Multivariate logistic regression of births at SCUH demonstrated a significantly higher chance of birth at term during both the lockdown period April–May 2020 (odds ratio (OR) 1.81, 95% CI 1.17, 2.79; *P* = 0.007) and post‐lockdown period June–July 2020 (OR 2.01, 95% CI 1.27, 3.18; *P* = 0.003), when compared with antecedent calendar‐matched periods in 2018–2019 (Supplementary Material).

All births at SCUH and in the state of Queensland overall are summarised in Table 1. During lockdown, the prevalence of PTB at SCUH was 5.5% (30/547), compared to 9.1% (100/1095) during the same period in previous years, representing a 40.0% relative reduction (*P* = 0.016). This reduction in PTB at SCUH appeared to be driven by moderate‐to‐late PTB (32 + 0–36 + 6 weeks). The prevalence of moderate‐to‐late PTB was 4.6% (25/547) during lockdown compared to 7.5% (82/1095) in previous years, a 39.0% relative reduction (*P* = 0.034). In contrast, the prevalence of PTB in Queensland overall was 8.6% (870/10 154) during lockdown compared to 8.9% (1871/20 939) in previous years, a nonsignificant 4.1% relative reduction in PTB (*P* = 0.33).

**Table 1:** Birth outcomes during the lockdown and post‐lockdown periods 2018–2020 at the Sunshine Coast University Hospital and in the state of Queensland overall

Birth outcome No./Total No. (%)	No lockdown Apr–May 2018–2019 (*n* = 22 034)	Lockdown Apr–May 2020 (*n* = 10 701)	*P*	No lockdown Jun–Jul 2018–2019 (*n* = 21 568)	Post‐lockdown Jun–Jul 2020 (*n* = 10 686)	*P*
Total preterm births/Total births						
SCUH^†^	100/1095 (9.13)	30/547 (5.48)	**0.016**	80/1130 (7.08)	26/524 (4.96)	0.12
QLD^‡^	1871/20 939 (8.94)	870/10 154 (8.57)	0.33	1865/20 438 (9.13)	921/10 162 (9.06)	0.87
Extreme preterm (20 + 0–27 + 6)						
SCUH	12/1095 (1.10)	3/547 (0.55)	0.41	5/1130 (0.44)	5/524 (0.95)	0.21
QLD	193/20 939 (0.92)	113/10 154 (1.11)	0.11	166/20 438 (0.81)	87/10 162 (0.86)	0.69
Very preterm (28 + 0–31 + 6)						
SCUH	6/1095 (0.55)	2/547 (0.37)	>0.99	3/1130 (0.27)	1/524 (0.19)	>0.99
QLD	163/20 939 (0.78)	79/10 154 (0.78)	>0.99	191/20 438 (0.93)	77/10 162 (0.76)	0.12
Moderate/late preterm (32 + 0–36 + 6)						
SCUH	82/1095 (7.49)	25/547 (4.57)	**0.034**	72/1130 (6.37)	20/524 (3.82)	**0.045**
QLD	1515/20 939 (7.24)	678/10 154 (6.68)	0.09	1508/20 438 (7.38)	757/10 162 (7.45)	0.84

^†^ SCUH, Sunshine Coast University Hospital.

^‡^ QLD: State of Queensland, Australia.

Bold indicates significance of *P* < 0.05.

After lockdown restrictions eased, the prevalence of PTB at SCUH was 5.0% (26/524) during the post‐lockdown period compared to 7.1% (80/1130) in previous years, a nonsignificant 29.9% relative reduction in PTB (*P* = 0.12). This reduction appeared to be driven by moderate‐to‐late PTB, as the prevalence in this group was 3.8% (20/524) during post‐lockdown, compared to 6.4% (72/1130) in previous years, a 40.0% relative reduction (*P* = 0.045). In the state of Queensland overall, there was no significant difference in PTB observed in the post‐lockdown period compared to previous years.

Table 2 outlines the perinatal characteristics of births at SCUH between April 1–July 31, 2018–2020. Most pregnant women were aged 20–40 years and only 4.3% of women were in the higher risk age groups for PTB of ≤20 years or ≥40 years.^18^ There was a general increase in BMI in both the lockdown and post‐lockdown groups in 2020 when compared to 2018–2019, consistent with the rising prevalence of obesity.^17^ At SCUH during lockdown, we observed a 29.5% relative decrease in emergency caesarean sections (*P* < 0.01) and concurrent 41.5% relative increase in instrumental vaginal births (*P* < 0.01) when compared to previous years.

**Table 2:** Perinatal characteristics at the Sunshine Coast University Hospital 2018–2020

Characteristic No./Total No. (%)	No lockdown Apr–May 2018–2019	Lockdown Apr–May 2020	*P*	No lockdown Jun–Jul 2018–2019	Post‐lockdown Jun–Jul 2020	*P*
Maternal age,^†^ median (IQR)	31 (27–34)	30 (26.5–34)	>0.99	30 (26–34)	31 (27–34)	0.78
Age under 20 years	23/1095 (2.10)	8/547 (1.46)	0.40	15/1130 (1.33)	4/524 (0.76)	0.46
Age 20–40 years	1045/1095 (95.43)	527/547 (96.35)	0.90	1078/1130 (95.40)	505/524 (96.38)	0.89
Age over 40 years	27/1095 (2.47)	12/547 (2.19)	0.74	37/1130 (3.27)	15/524 (2.86)	0.67
Body mass index,^†^ median (IQR)	27 (24–58)	29 (25–54)	**<0.01**	28 (24–60)	29 (25–58)	**0.030**
Underweight, <18	30/1095 (2.74)	10/547 (1.83)	0.27	25/1130 (2.21)	3/524 (0.57)	**0.022**
Healthy weight, 18–25	603/1095 (55.07)	234/547 (42.78)	**<0.01**	570/1130 (50.45)	242/524 (46.18)	0.34
Overweight, 25–30	296/1095 (27.03)	165/547 (30.16)	0.32	304/1130 (26.90)	150/524 (28.63)	0.58
Obese >30	142/1095 (12.97)	113/547 (20.66)	**<0.01**	194/1130 (17.17)	98/524 (18.70)	0.52
Unknown	24/1095 (2.19)	25/547 (4.57)	**<0.01**	37/1130 (3.27)	31/524 (5.92)	**0.016**
Parity						
Nulliparous	425/1095 (38.81)	218/547 (39.85)	0.79	448/1130 (39.64)	202/524 (38.55)	0.78
Primiparous	384/1095 (35.07)	190/547 (34.74)	0.93	375/1130 (33.19)	183/524 (34.92)	0.63
Multiparous	286/1095 (26.12)	139/547 (25.41)	0.81	307/1130 (27.17)	139/524 (26.53)	0.84
Mode of delivery						
Spontaneous vaginal births	661/1095 (60.37)	318/547 (58.14)	0.66	651/1130 (57.61)	278/524 (53.05)	0.35
Instrumental vaginal births	116/1095 (10.59)	82/547 (14.99)	**<0.01**	167/1130 (14.78)	85/524 (16.22)	0.46
Vacuum‐assisted birth	96/1095 (8.77)	68/547 (12.43)	**0.036**	143/1130 (12.65)	69/524 (13.17)	0.80
Forceps‐assisted birth	19/1095 (1.74)	14/547 (2.56)	0.27	23/1130 (2.04)	16/524 (3.05)	0.22
Vacuum + forceps‐assisted birth	1/1095 (0.09)	0/547 (0.00)	>0.99	1/1130 (0.09)	0/524 (0.00)	>0.99
Elective caesareans	125/1095 (11.41)	79/547 (14.44)	0.12	136/1130 (12.04)	72/524 (13.74)	0.39
Emergency caesareans	193/1095 (17.63)	68/547 (12.43)	**<0.01**	176/1130 (15.58)	89/524 (16.98)	0.47
Emergency caesarean, in labour	156/1095 (14.24)	55/547 (10.05)	**0.034**	152/1130 (13.45)	79/524 (15.08)	0.44
Emergency caesarean, no labour	37/1095 (3.38)	13/547 (2.38)	0.28	24/1130 (2.12)	10/524 (1.91)	0.78

† *P*‐values calculated with the Mann–Whitney *U*‐test. Unmarked *P*‐values calculated using χ^2^ or Fisher's exact test, where appropriate.

Bold indicates significance of *P* < 0.05. IQR, interquartile range.

Although the overall prevalence of PTB decreased during lockdown at SCUH, there was no change identified in the ratio between spontaneous or iatrogenic (medically indicated) PTB during lockdown or post‐lockdown (Table 3). A nonsignificant increase in stillbirth was observed during lockdown (*P* = 0.09) and post‐lockdown (*P* = 0.08) when compared to previous years at SCUH, although the numbers were low. There was no change in the prevalence of late terminations, compared to previous years at SCUH.

**Table 3:** Birth outcomes and proposed aetiologies for preterm birth during the lockdown and post‐lockdown periods 2018–2020 at the Sunshine Coast University Hospital

Birth outcome No./Total No. (%)	No lockdown Apr–May 2018–2019	Lockdown Apr–May 2020	*P*	No lockdown Jun–Jul 2018–2019	Post‐lockdown Jun–Jul 2020	*P*
Spontaneous live preterm birth	52/100 (52.00)	16/30 (53.33)	0.94	50/80 (62.50)	11/26 (42.31)	0.33
Preterm premature rupture of membranes	26/100 (50.00)	8/30 (26.67)	0.96	28/80 (35.00)	7/26 (26.92)	0.58
Preterm labour and birth	16/100 (30.77)	6/30 (20.00)	0.67	18/80 (22.50)	4/26 (15.38)	0.78
Multiple pregnancy	10/100 (19.23)	2/30 (6.67)	>0.99	4/80 (5.00)	0/26 (0.00)	0.57
Iatrogenic live preterm birth	39/100 (39.00)	11/30 (36.67)	0.88	24/80 (30.00)	8/26 (30.77)	0.96
Intrauterine growth restriction	10/100 (10.00)	3/30 (10.00)	>0.99	4/80 (5.00)	1/26 (3.85)	>0.99
Hypertension	8/100 (8.00)	2/30 (6.67)	>0.99	5/80 (6.25)	2/26 (7.69)	>0.99
Multiple pregnancy	17/100 (17.00)	4/30 (13.33)	0.79	10/80 (12.50)	4/26 (15.38)	0.75
Other maternal comorbidities	2/100 (2.00)	1/30 (3.33)	0.55	2/80 (2.50)	1/26 (3.85)	>0.99
Fetal distress	1/100 (1.00)	1/30 (3.33)	0.42	2/80 (2.50)	0/26 (0.00)	>0.99
Placental abruption or antepartum haemorrhage	1/100 (1.00)	0/30 (0.00)	>0.99	1/80 (1.25)	0/26 (0.00)	>0.99
Stillbirth ≥20 weeks	2/100 (2.00)	3/30 (10.00)	0.09	3/80 (3.75)	4/26 (15.38)	0.08
Termination ≥20 weeks	7/100 (7.00)	0/30 (0.00)	0.35	3/80 (3.75)	3/26 (11.54)	0.18

## DISCUSSION

Australia enforced strict lockdowns to mitigate the spread of COVID‐19, reaching a stringency level of 71.3 in a 1‐to‐100 scale on the Oxford Government Response Tracker.^19^ Residents were not permitted to leave their home except for essential reasons.^3^ At SCUH in Queensland Australia, there was a significantly higher chance of birth at term during lockdown and post‐lockdown, when compared to previous years. During lockdown, a 40.0% relative reduction in the prevalence of PTB was observed at SCUH. This finding is consistent with similar studies worldwide which reported a significantly lower prevalence of PTB during lockdown.^6^, ^7^, ^9^, ^11^, ^12^ In contrast to the reduction in PTB observed at SCUH, there was a nonsignificant decrease in PTB observed in the state of Queensland overall during lockdown. Our study and another study from Brisbane,^6^ both metropolitan areas in Queensland, observed reductions in PTB. However, in our study, the reduction in PTB was not observed for the state of Queensland overall. It is possible the effect of lockdown on PTB was more apparent in metropolitan areas, although this requires further investigation. At SCUH, the reduction in the prevalence of PTB during lockdown was driven by moderate‐to‐late PTB, in line with other studies.^6^ From June 2020, severe restrictions were lifted, although mild restrictions remained. In the post‐lockdown period, the reduction in moderate‐to‐late PTB was sustained, which may have been due to ongoing mild restrictions, or that altered maternal behaviours enforced during lockdown persisted even after restrictions eased.

The obstetric antecedents of PTB include delivery for materno‐fetal indications (iatrogenic), spontaneous preterm labour, and preterm premature rupture of membranes.^18^ In high‐income countries, 30.0% of PTB are iatrogenic, while 70.0% are spontaneous.^18^ The aetiology of most spontaneous PTB is unknown, the most common cause identified is intrauterine infection, inflammation, and placental anomalies.^18^ We did not observe a significant difference in the ratio between spontaneous or iatrogenic PTB, despite an overall reduction in PTB during lockdown, compared to previous years. However, other studies from Australia^6^ and Israel^13^ reported a reduction in iatrogenic PTB during lockdown.

In Western Australia, Newnham *et al*. demonstrated a state‐wide reduction in PTB through a comprehensive PTB prevention program.^20^ This likely contributed to PTB reduction throughout Australia since 2015, although we would expect this to remain consistent throughout our study period from 2018 to 2020. During lockdown, maternal behaviours were changed as a result of restrictions and public health messaging, thus women likely had increased health awareness. During lockdown, Australia noted improved air quality,^21^ known to impact PTB.^22^ Although the causality between PTB and work remains controversial, with more women working from home or unemployed, there may have been more time for sleep, exercise, and healthy eating.^14^ It is possible that PTB rates may have been reduced by other factors including adapted antenatal care that COVID‐safe practices required, women may have birthed at different hospitals during lockdown, or other causes not yet delineated. In contrast, it is important to recognise the negative impacts of lockdown for some women, as there was a rise in domestic violence.^23^

A decrease in the transmission of infections, such as influenza, a known risk factor for PTB^24^ may have contributed to reductions in PTB during lockdown. Pregnant women have an increased risk of severe influenza and PTB, thus pregnant women are recommended to receive the influenza vaccine, which reduces the risk of influenza for the woman, the risk of PTB, and confers immunity to the newborn.^24^

In Queensland, Australia, maternal influenza vaccination uptake has been suboptimal with 42.0% of women receiving the vaccine in 2018, despite it being encouraged and free‐of‐charge.^17^ We also recognise that vaccination uptake is lower in people living in regional and remote areas; a large proportion of Queenslanders live outside cities.^17^ During the pandemic, the Australian government introduced measures to increase the uptake of influenza vaccination and emphasised the importance of pregnant women receiving the vaccine.^25^ Influenza vaccination uptake in Australia increased by 108.6%, from 3.5 million in 2018 to 7.3 million doses by May 2020 during lockdown, which may have been attributed to effective public health messaging and increased health awareness during the pandemic.^26^ In 2020, Australia noted the lowest influenza rate of the decade, eight times lower than the five‐year average,^27^ likely as a result of the high vaccination uptake and reduced transmission of infections during lockdown. Both high influenza vaccination uptake and low influenza infection rates may have contributed to the reduction observed in PTB, although this requires further study.

During lockdown at SCUH, there was a 29.5% relative decrease in emergency caesarean sections and concurrent 41.5% relative increase in instrumental vaginal births. This trend did not extend into the post‐lockdown period. The relationship between decreased emergency caesareans and increased instrumentals during lockdown is unclear. A similar study from Japan^11^ demonstrated a comparable reduction and a British study^10^ showed no change in caesarean sections during lockdown. Research into provision of maternity care during lockdown was beyond the scope of this study but would be of great interest.

At SCUH, there was a nonsignificant increase in stillbirth during lockdown and post‐lockdown. Other studies demonstrated a wide variation in stillbirth rates during lockdown; studies from Australia,^6^, ^12^ England,^28^, ^29^ and Israel^13^ showed no change, while studies from England^10^ and Italy^8^ demonstrated increased stillbirth rates during lockdown. No change was observed in the prevalence of late terminations during lockdown, compared to previous years at SCUH. In Australia during lockdown, terminations were classed as urgent medical treatment, so direct access was not impaired, although restrictions on movement and indirect effects for vulnerable people may have been amplified during lockdown.^30^ Further research is required into stillbirth and termination care during times of altered health service provision.

Our study is the first to examine state‐wide PTB data during the COVID‐19 pandemic in Australia. This allowed for comparison of one health region to a large state overall, including metropolitan, regional, and rural areas. This paper is one of only three studies from Australia to determine the prevalence of PTB during the pandemic. Further, our study is the first paper to highlight the possible association between reduced PTB during lockdown and increased influenza vaccination rates in Queensland, Australia. For births at SCUH, we analysed perinatal characteristics including maternal demographics and comorbidities, mode of delivery, and birth outcomes including spontaneous and iatrogenic PTB, stillbirth, and late terminations.

Our study has limitations, which should be considered when interpreting results. As this study was observational, it was not possible to account for unmeasured confounding variables. Although detailed data were collected on all births at SCUH, state‐wide data for Queensland was limited to birth outcomes and gestational age, as per the predetermined data collection format. As our health service expanded from April 2017 (SCUH), analysis was only completed for two years prior. We acknowledge longer retrospective analysis would be preferable to further compare year‐by‐year variations.

Additional research is required from ethnically, geographically, and socioeconomically diverse regions and high‐risk groups for PTB such as Aboriginal and Torres Strait Islander women in Australia. Qualitative analysis of maternal behaviours during lockdown was beyond the scope of this study but would be highly informative.

There is a link between lockdown and a reduction in the prevalence of PTB on the Sunshine Coast. The cause is speculative at present, although increased influenza vaccination rates, decreased transmission of infections, and improved air quality may have been favourable in reducing preterm birth during lockdown. Further research is needed to determine a causal link and assess if this can be translated into management which provides a sustained reduction in PTB and its associated morbidity and mortality.

## AUTHOR CONTRIBUTIONS

BJ, TS, EW conceptualised and designed the study. BJ and TS obtained and analysed the initial data. BJ, TS, CA, EW analysed the final data. BJ and TS drafted the initial manuscript. BJ, TS, CA, EW critically reviewed and revised the final manuscript. EW supervised the project.

## DISCLAIMER

The views expressed in this paper are those of the authors, and are not representative of other bodies such as the Sunshine Coast University Hospital, Griffith University, the University of Queensland, North West Anglia Healthcare NHS Trust, Cairns Hospital, Perinatal Data, Queensland Government, Department of Health, or the Preterm Infants Parents Association.

## Supporting information


**Table S1** Univariate analysis: logistic regression using term (0 = preterm, 1 = term) as the dependent (outcome) variable at the Sunshine Coast University Hospital from 2018 to 2020.
**Table S2** Multivariate analysis: logistic regression using term (0 = preterm, 1 = term) as the dependent (outcome) variable at the Sunshine Coast University Hospital from 2018 to 2020.

## Data Availability

The authors confirm that the data supporting the findings of this study are available within the article and its Supplementary Materials.
