# Supplementary material for: Reduction in preterm birth rates during and after the COVID‐19 lockdown in Queensland Australia
Source: Aust N Z J Obstet Gynaecol. 2022 May 17:10.1111/ajo.13538. Online ahead of print. doi: 10.1111/ajo.13538 (PMC9348165; doi:10.1111/ajo.13538)
Supplement: Supplementary file 1 — Table S1 Univariate analysis: logistic regression using term (0 = preterm, 1 = term) as the dependent (outcome) variable at the Sunshine Coast University Hospital from 2018 to 2020. Table S2 Multivariate analysis: logistic regression using term (0 = preterm, 1 = term) as the dependent (outcome) variable at the Sunshine Coast University Hospital from 2018 to 2020. [file AJO-9999-0-s001.docx]

**ONLINE SUPPORTING INFORMATION**

**Percent change calculation**

Percent change (relative decrease or increase) was calculated to assess for any difference in incidence of birth outcomes between time periods [percent change = [((final value–initial value)/initial value)x100].

**Supplementary table 1.** Univariate analysis: logistic regression using term (0=preterm, 1=term) as the dependent (outcome) variable at the Sunshine Coast University Hospital from 2018–2020.

| **Predictor (explanatory) variable** | **Codes** | **Odds Ratio (95% CI)** | **P** |
| --- | --- | --- | --- |
| **Demographics** |  |  |  |
| Maternal age (years) | – | 0.99 (0.97, 1.02) | 0.484 |
| Maternal BMI (kg.m^-2^) | – | 0.99 (0.98, 1.01) | 0.097 |
| **Parity** |  |  |  |
| Nulliparous vs Multiparous | 0 vs 2 | 0.90 (0.76, 1.06) | 0.195 |
| Primiparous vs Multiparous | 1 vs 2 | 0.76 (0.55, 1.07) | 0.118 |
| **COVID-19 periods** |  |  |  |
| Pre (Apr–May 2018–2019) vs Pre (Jun–Jul 2018–2019) | 1 vs 2 | 1.32 (0.97, 1.79) | 0.077 |
| Pre (Apr–May 2018–2019) vs Lockdown (Apr–May 2020) | 1 vs 3 | 1.31 (1.07, 1.62) | **0.011** |
| Pre (Jun–Jul 2018–2019) vs Post-lockdown (Jun–Jul 2020) | 2 vs 4 | 1.21 (0.96, 1.52) | 0.104 |

**Bold** indicates significance of *P* value <0.05

P values <0.20 were included in multivariate logistic regression

**Supplementary table 2.** Multivariate analysis: logistic regression using term (0=preterm, 1=term) as the dependent (outcome) variable at the Sunshine Coast University Hospital from 2018–2020.

| **Variable** | **Odds Ratio (95% CI)** | **P** |
| --- | --- | --- |
| **Demographics** |  |  |
| Maternal age (years) | 1.01 (0.97, 1.05) | 0.695 |
| Maternal BMI (kg.m^-2^) | 0.99 (0.99, 1.01) | 0.100 |
| **Parity** |  |  |
| Primiparous | 1.04 (0.71, 1.51) | 0.858 |
| Multiparous | 0.78 (0.47, 1.28) | 0.324 |
| **COVID-19 periods** |  |  |
| Lockdown (Apr–May 2020) | 1.81 (1.17, 2.79) | **0.007** |
| Post-lockdown (Jun–Jul 2020) | 2.01 (1.27, 3.18) | **0.003** |

**Bold** indicates significance of P value <0.05

**Interpretation:**

An Odds Ratio (OR) < 1.00 decreases the odds of Term birth. An Odds Ratio (OR) > 1.00 increases the odds of Term birth. Only births during the lockdown and post-lockdown were significant predictors.

**Summary:** Within the Sunshine Coast University Hospital, there was a significantly higher chance of birth at term during both the Lockdown (OR 1.81) and Post-lockdown (OR 2.01) periods.
